# Transfemoral versus transapical approach for transcatheter aortic valve implantation: hospital outcome and risk factor analysis.

**DOI:** 10.1186/s13019-017-0638-9

**Published:** 2017-09-06

**Authors:** Enrico Ferrari, Eric Eeckhout, Sanjiv Keller, Olivier Muller, Piergiorgio Tozzi, Denis Berdajs, Ludwig Karl von Segesser

**Affiliations:** 1 Department of Cardiac Surgery, Cardiocentro Ticino Foundation, Via Tesserete 48, 6900 Lugano, Switzerland; 2 Cardiovascular Research Unit, University Hospital, Lausanne, Switzerland; 3 Cardiology Unit, University Hospital, Lausanne, Switzerland; 4 Cardiac Surgery Unit, University Hospital of Basel, Basel, Switzerland

**Keywords:** Transcatheter aortic valve implantation, Aortic valve stenosis, Transfemoral aortic valve implantation, Transapical aortic valve implantation

## Abstract

**Background:**

Transcatheter aortic valve implantation is indicated in high-risk patients with aortic stenosis. We compared the clinical outcome of 180 consecutive patients who underwent transapical (TA) and transfemoral (TF) procedures in a single centre.

**Methods:**

Ninety consecutive TA (TA-group) and 90 consecutive TF (TF-group) were performed from 2009 to 2014. Clinical variables were prospectively collected and retrospectively analysed for hospital outcomes and to identify risk factors for hospital mortality, vascular complications and stroke.

**Results:**

Mean age was 80 ± 8.5 and 83 ± 8.4 years, in the TA and TF-group, respectively. TA-group presented higher prevalence of comorbidities: more vascular disease (79% vs 22%, *p* < 0.001), chronic pulmonary disease (32% vs 10%, *p* < 0.001), previous vascular surgery (14% vs 4%, *p* = 0.039), coronary disease (60% vs 40%, *p* = 0.007), and previous cardiac surgery (28% vs 17%, *p* = 0.073). Logistic Euroscore was 36 ± 15% in the TA-group and 25 ± 14% in the TF-group (*p* < 0.001), but hospital mortality was similar (TA:9%, TF:10%, *p* = 0.799). Access-related vascular complications occurred more often in transfemoral patients (TA:3%, TF:11%, *p* = 0.081) while major bleeding (TA:3%, TF:4%, *p* = 1) and stroke (TA:2%, TF:3%, *p* = 1) were equally distributed. Postoperative renal failure and dialysis were associated with impaired neurological outcome (*p* = 0.035 and *p* = 0.020, respectively). Mild to severe paravalvular leak was more prevalent in transfemoral patients (TA:5%, TF:25%, *p* < 0.001).

**Conclusions:**

In our experience, the TA and TF-group presented different risk profiles but mortality rate and adverse neurological outcome had a similar incidence. The transfemoral approach carried more vascular complications and paravalvular leaks but last-generation devices will improve this outcome.

## Background

Aortic stenosis is the most common acquired heart valve disease in the adult and the surgical aortic valve replacement (SAVR) remains the treatment of choice with good outcomes and long-term results [1–4]. However, patients with comorbidities carry a higher surgical risk and, therefore, they might benefit from recently developed minimally invasive techniques and technologies. Since 2007, the transcatheter aortic valve implantation (TAVI) has become a widely-accepted alternative to open heart surgery in patients with high-risk profiles and, so far, more than 200′000 procedures have already been performed worldwide, mostly with the CoreValve™ (Medronic, Minneapolis, MN) and the SAPIEN™ (Edwards Lifesciences, Irvine, CA) valve families with good hospital and mid-term results [5–8]. The two arms of the PARTNER trial have proven the safety and efficacy of TAVI in inoperable and high-risk patients with superior results when compared to medical treatment and non-inferior results when compared to standard surgery [7, 8]. Moreover, recently published studies have also shown good valve hemodynamic parameters in early and mid-term follow-ups and in intermediate-risk patients [9–15].

Alternative access routes have been explored but the two most popular are still the transapical (TA) and the transfemoral (TF): however, patient attribution to these accesses is still questionable in absence of a severe peripheral vascular disease. We compared the outcome of our first 180 consecutive TA and TF TAVI patients and we identified risk factors for postoperative hospital mortality, vascular complications and stroke.

## Methods

In the database, we identified the first 90 TA-TAVI cases (TA-group) and the first 90 TF-TAVI cases (TF-group) performed from 2009 to 2014. In our institution, TA-TAVI was performed since 2009 while TF-TAVI were scheduled since 2010. During the same period of time, only few transaortic TAVI have been performed and they are not included in the study.

Clinical variables were prospectively collected in the hospital database and then retrospectively analysed to compare the two groups and to identify risk factors for hospital mortality (30 days mortality or within hospital stay), neurological events (stroke) and vascular complications (major vascular complications including access-site related complications, aortic dissections, aortic root rupture and tamponade due to ventricular rupture) using the VARC-2 definitions described elsewhere [16]. All patients signed informed consents for the index procedure, for the use of clinical data in medical research and for clinical data analysis (Patients included in the Swiss TAVI Registry approved by the Ethics Committee of the Vaud State in Switzerland).

### Patients selection criteria

Patients suffering from severe symptomatic aortic valve stenosis with concomitant comorbidities were studied for inclusion in the TAVI program. Standard inclusion criteria for TAVI were used to identify good candidates and the Logistic Euro-SCORE was calculated for all patients to predict hospital mortality. In order to proceed with transcatheter interventions the final decision came from the hospital Heart-Team, in particular concerning patients not fulfilling standard criteria (i.e. younger patients with severe liver disease or patients with porcelain aortas).

Important exclusion criteria for both TA and TF approach were the presence of concomitant severe valvular or coronary disease not suitable for percutaneous procedure, severe left ventricular dysfunction with ejection fraction below 20% and severe patient frailty evaluated by the Heart-Team, which consists of a cardiologist, a surgeon (both coordinators for the TAVI program), an anaesthesiologist, a radiologist and a geriatrician.

**Fig. 1:**
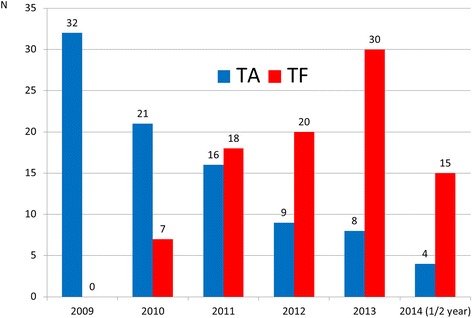
Transapical and transfemoral TAVI distribution in our hospital during the study period

An important observation is that during the study period, transapical and transfemoral procedures were not equally distributed (Fig. 1). This was due to the access-site selection process performed by the Heart-Team that embraced, during the years, the launch of new “low-profile” transfemoral devices leading to more valves implanted transfemorally.

All patients enrolled in the TAVI program underwent complementary trans-thoracic echocardiogram, coronary angiogram and three-dimensional cardiovascular computed tomography scan (3D CT-scan) to analyse alternative access routes and to determine the valve size. Peripheral vascular access routes smaller than 6 mm diameter or presenting severe annular calcifications were not considered good access sites for transfemoral TAVR, such as the presence of abdominal aorta aneurysms and vessel tortuosity. In these cases, TA was considered the best option. Pre-dilation of small ilio-femoral arteries was never attempted. The aortic valve annulus was assessed by injected aortic CT-scan and trans-oesophageal echocardiography using the diameter, the area and the perimeter for the stent-valve sizing. Patients with chronic kidney failure underwent CT-scan and coronary angiograms with low-dose of contrast.

### Ta-Tavi

TA-TAVI were performed under general anaesthesia through a left antero-lateral mini-thoracotomy at fifth intercostal space. Intraoperative imaging included trans-oesophageal echocardiography to confirm the valve size and fluoroscopy. The apexes were prepared with concentric reinforced purse-string sutures and the devices were the Sapien™ (2009–2010), the Sapien™ XT (2011–2013) and the Sapien™ 3 (2014) valves (Edwards Lifesciences, Irvine, CA). At the beginning of our series, complication-free patients used to be transferred intubated to the intensive care unit while, after getting more experienced, patients were rapidly extubated in the cath-lab and transferred to the intermediate care unit. As previously reported, echo-guided TA-TAVI were performed without contrast injections [17].

### Tf-Tavi

Patients with good vascular access underwent TF-TAVI under general anaesthesia. According to our policy, the vascular access was surgically performed with a 3 cm skin incision at the groin in order to puncture the femoral artery under direct vision and prevent collateral damage. Cardiac imaging was similar to TA-TAVI while the devices were the Sapien™ valves, as mentioned above, but also the Medtronic CoreValve™ (Medtronic inc, Minneapolis, MN). All complication-free patients were extubated in the cath-lab and transferred to the intermediate care unit.

### Statistical analysis

Statistical analysis was performed using R (version 3.2.2). Continuous variables are summarized as mean ± Standard Deviation and a t-test is used to compare the two groups. Categorical variables are presented as numbers and proportions (%) and a χ^2^ test or a Fisher exact test is used to compare the two groups. Selected categorical and continuous variables were analysed as risk-factors for hospital mortality (death occurring within 30 days or during the same hospitalization), disabling neurological complications and major vascular complications, using univariate logistic regression. A *p-*value below 0.05 was considered statistically significant.

## Results

Baseline clinical characteristics are described in Tables 1 and 2. Mean age was 80 ± 8.5 and 83 ± 8.4 years, in the TA and TF-group, respectively (*p* = 0.014), while the sex distribution was similar (50% male patients in the TA-group and 41% in the TF-group, *p* = 0.231).

**Table 1:** Demographics, symptoms, risk factors

	Overall (*N* = 180)	TA-TAVI (*N* = 90)	TF-TAVI (*N* = 90)	p
Mean age (years)	82 ± 8.6	80 ± 8.5	83 ± 8.4	0.014
Men	82 (46%)	45 (50%)	37 (41%)	0.231^a^
COPD	38 (21%)	29 (32%)	9 (10%)	<0.001^a^
Peripheral vascular disease	91 (51%)	71 (79%)	20 (22%)	<0.001^a^
Previous vascular surgery	17 (9%)	13 (14%)	4 (4%)	0.039
Coronary disease	90 (50%)	54 (60%)	36 (40%)	0.007^a^
Previous coronary surgery	27 (15%)	19 (21%)	8 (9%)	0.022^a^
Previous cardiac surgery	40 (22%)	25 (28%)	15 (17%)	0.073^a^
Previous coronary angioplasty/stenting	28 (16%)	13 (14%)	15 (17%)	0.681^a^
Hypertension	117 (65%)	56 (62%)	61 (68%)	0.435^a^
Chronic renal failure	75 (42%)	38 (42%)	37 (41%)	0.880^a^
Dialysis	7 (4%)	4 (4%)	3 (3%)	1
Previous stroke	20 (11%)	11 (12%)	9 (10%)	0.635^a^
Diabetes (insulin)	30 (17%)	18 (20%)	12 (13%)	0.230^a^
Liver disease (CHILD score)	5 (3%)	3 (3%)	2 (2%)	1
pacemaker implantation	21 (12%)	9 (10%)	12 (13%)	0.486^a^
Chest radiotherapy	16 (9%)	7 (8%)	9 (10%)	0.600^a^
Preoperative critical state	15 (8%)	13 (14%)	2 (2%)	0.005
Porcelain aorta	12 (13%)	12 (13%)	0 (0%)	1
logistic Euro-SCORE (%)	31 ± 16	36 ± 15	25 ± 14	<0.001

Data presented as mean ± SD or N (%)

^a^ Chi2 value, otherwise it’s a Fischer test or a T-Test value

*COPD* Chronic Obstructive Pulmonary Disease

**Table 2:** Preoperative imaging and valve hemodynamic

	Overall (*N* = 180)	TA-TAVI (*N* = 90)	TF-TAVI (*N* = 90)	p
Transaortic valve gradient (mmHg)	67 ± 26	62 ± 25	71 ± 28	
Mean aortic valve area (cm^2^)	0.9 ± 3.4	1.2 ± 4.8	0.7 ± 0.2	
Indexed aortic valve area (cm^2^/m^2^)	0.4 ± 0.1	0.4 ± 0.1	0.4 ± 0.1	
Mean left ventricular ejection fraction (%)	54 ± 13	52 ± 12	56 ± 13	
> 50%	101 (56%)	42 (47%)	59 (66%)	0.008*
30–50%	70 (39%)	42 (47%)	28 (32%)	
< 30%	8 (4%)	6 (6%)	2 (2%)	
Pulmonary hypertension	101 (56%)	48 (53%)	53 (59%)	0.453*
Mean aortic annulus diameter measured with CT-scan (mm)	23 ± 2.3	23 ± 2.5	24 ± 2.1	
Mean aortic annulus diameter measured with TOE (mm)	23 ± 2.2	22 ± 2	22 ± 2.4	
Mean distance: annulus-left coronary ostium (mm)	13 ± 2.9	12 ± 2	14 ± 3.2	
Mean distance: annulus-right coronary ostium (mm)	134 ± 4	12 ± 3	15 ± 4.4	

Data presented as mean ± SD or N (%). * Chi2 value

*CT* Computed Tomography, *TOE* Transoesophageal echocardiography

TA-group presented higher prevalence of comorbidities: more vascular disease (TA:79%, TF:22%, *p* < 0.001), chronic pulmonary disease (TA:32%, TF:10%, *p* < 0.001), higher prevalence of previously performed vascular surgery (TA:14%, TF:4%, *p* = 0.039), higher coronary disease (TA:60%, TF:40%, *p* = 0.007), more previously performed coronary surgery (TA:21%, TF:9%, *p* = 0.022) and more previously performed cardiac surgery (TA:28%, TF:17%, *p* = 0.073). Thirteen percent of patients in the TA-group had a porcelain aorta, versus none in the TF-group. More patients with critical preoperative state were included in the TA-group (TA:14%, TF:2%, *p* = 0.005) and the logistic Euro-SCORE was higher in the TA-group (36 ± 15% for the TA-group compared to 25 ± 4% for the TF-group; *p* < 0.001).

Successful implantation rate was 100% with longer procedural time in the TF-group (TA:98 ± 33 min, TF:127 ± 56 min, *p* < 0.001). Twelve patients had redo valve-in-valve for degenerated aortic bioprosthesis, while seven patients received two stent-valves because of a stent-valve malpositioning or migration of the previously implanted stent-valve.

Mean valve size and valve size distribution are listed in Table 3.

**Table 3:** Procedural data

	Overall (*N* = 180)	TA-TAVI (*N* = 90)	TF-TAVI (*N* = 90)	p
Sapien™ and Sapien XT™	146 (81%)	86 (96%)	60 (67%)	
Sapien 3™	8 (4%)	4 (4%)	4 (4%)	
CoreValve™	26 (29%)	NA	26 (29%)	
Valve-in-valve in degenerated bioprosthesis	12 (7%)	7 (8%)	5 (6%)	0.550^a^
Bailout valve-in-valve for migration or malpositioning	7 (4%)	3 (3%)	4 (4%)	1
Mean valve size (mm)	25 ± 2.1	25 ± 1.9	25 ± 2.2	
Valve size distribution				
23 mm	75 (42%)	40 (44%)	35 (39%)	
26 mm	77 (43%)	43 (48%)	44 (49%)	
29 mm	14 (8%)	7 (8%)	7 (8%)	
31 mm	4 (4%)	0 (0%)	4 (4%)	
Procedural time (min)	113 ± 48	98 ± 33	127 ± 56	<0.001

Data presented as mean ± SD or N (%)

^a^ Chi2 value, otherwise it’s a Fischer test or a T-Test value

**Fig. 2:**
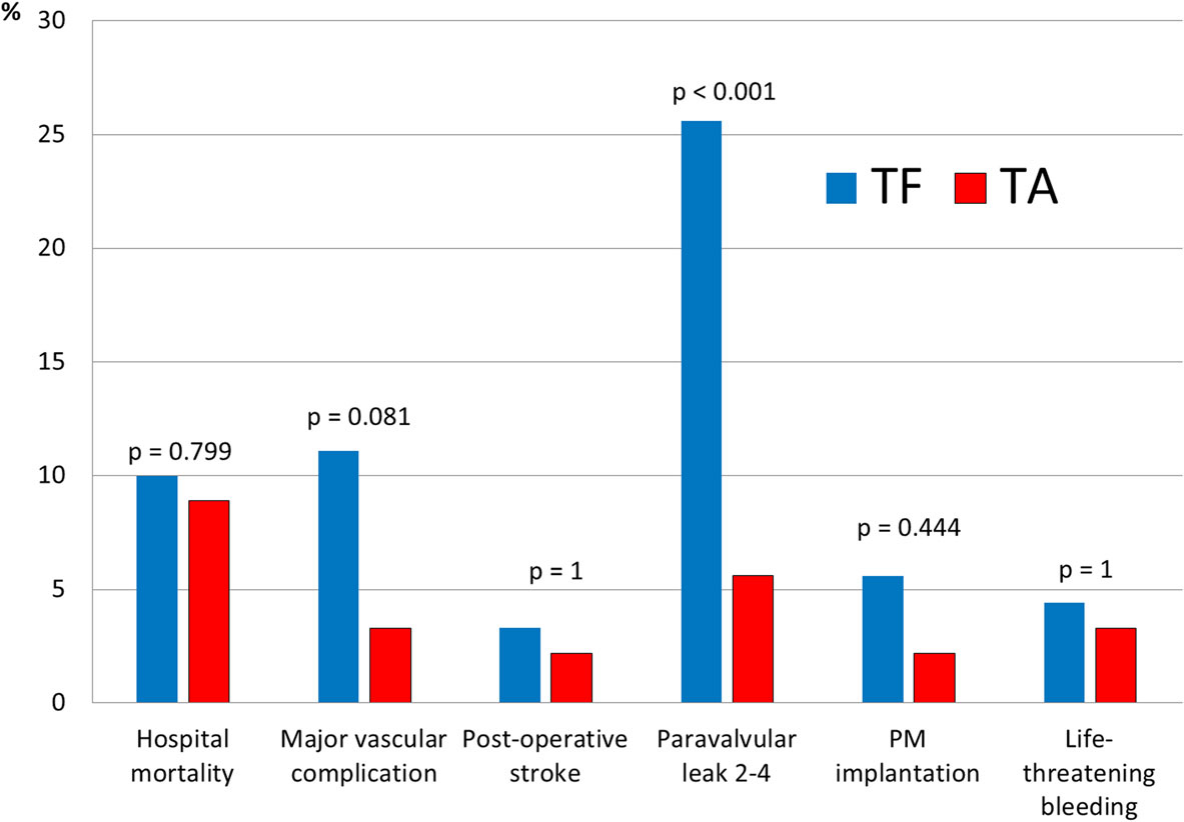
Postoperative complications of TA and TF TAVI according to the VARC-2 definitions

With regards to hospital mortality and complication, variables were analysed using VARC-2 definitions (Table 4). Mortality for the two groups was similar (TA:9%, TF:10%, *p* = 0.799) with a learning curve effect at the beginning of our experience. Main cause of death in TA-group was respiratory failure (3%), whereas in the TF-group was disabling stroke (3%). Major vascular complications (including access-related vascular complication, aortic dissection, acute leg ischemia and aortic rupture) occurred more often in the TF-group (TA:3%, TF:11%, *p* = 0.081) whereas major/life-threatening bleeding (including cardiac tamponade) (TA:3%, TF:4%, *p* = 1), disabling stroke (TA:2%, TF:3%, *p* = 1), and bailout valve-in-valve for valve migration or malpositioning (TA:3%, TF:4%, *p* = 1) were equally distributed (Fig. 2). Re-thoracotomy for bleeding was performed in three TA and a percutaneous pericardial drainage for tamponade was urgently performed in two TF without further surgical exploration. Three patients in TA-group required dialysis.

**Table 4:** Hospital outcome

	Overall (*N* = 180)	TA-TAVI (*N* = 90)	TF-TAVI (*N* = 90)	p
Hospital mortality	17 (9%)	8 (9%)	9 (10%)	0.799*
Cause of death				
Respiratory failure	3 (2%)	3 (3%)	0 (0%)	
Cardiac tamponade for annulus rupture	2 (1%)	0 (0%)	2 (2%)	
Valve migration	1 (1%)	0 (0%)	1 (1%)	
Myocardial infarction	1 (1%)	1 (1%)	0 (0%)	
Heart failure	1 (1%)	0 (0%)	1 (1%)	
Sudden death	1 (1%)	1 (1%)	0 (0%)	
Cardiac arrest	2 (1%)	0 (0%)	2 (2%)	
Life-threatening bleeding	1 (1%)	1 (1%)	0 (0%)	
Multiple organ failure	1 (1%)	1 (1%)	0 (0%)	
Disabling stroke	4 (2%)	1 (1%)	3 (3%)	
Complications				
Major vascular complications (including access related vascular complication, aortic rupture and dissection, leg ischemia)	13 (7%)	3 (3%)	10 (11%)	0.081
Valve migration	3 (2%)	1 (1%)	2 (2%)	1
Major/life-threatening bleeding (including cardiac tamponade for aortic rupture or ventricular tear)	7 (4%)	3 (3%)	4 (4%)	1
Disabling Stroke	5 (3%)	2 (2%)	3 (3%)	1
Coronary occlusion	1 (1%)	1 (1%)	0 (0%)	1
Bailout Sapien-in-Sapien for stent-valve migration or malpositioning	7 (4%)	3 (3%)	4 (4%)	1
Pneumonia	6 (3%)	5 (6%)	1 (1%)	0.211
Rethoracotomy for bleeding (TA)/pericardial drainage for tamponade (TF)	5 (3%)	3 (3%)	2 (2%)	1
Postoperative acute renal failure	4 (2%)	3 (3%)	1 (1%)	0.621
Dialysis	3 (2%)	3 (3%)	0 (0%)	0.246
New pacemaker for conduction abnormality	7 (4%)	2 (2%)	5 (6%)	0.444
Conversion to sternotomy	2 (1%)	2 (2%)	0 (0%)	0.497
Bailout cardiopulmonary bypass	4 (2%)	4 (4%)	0 (0%)	0.121
Early extubation	123 (68%)	40 (44%)	83 (92%)	<0.001*
Intensive Care Unit stay (days)	1.4 ± 4.1 (median:0)	2.6 ± 5.5 (median:1)	0.2 ± 0.7 (median:0)	<0.001
Hospital stay (days)	11.7 ± 9.1 (median:9)	13.9 ± 9.5 (median:10)	9.4 ± 8.2 (median:8)	<0.001
Postoperative peak gradient (mmHg)	17.6 ± 9.5	16.7 ± 9.4	18.5 ± 9.5	0.219
Postoperative mean gradient (mmHg)	9.5 ± 5.3	9.2 ± 5.1	9.7 ± 5.5	0.563
Paravalvular leak: mild to severe	28 (16%)	5 (6%)	23 (26%)	<0.001*
Trace of paravalvular leak	48 (27%)	15 (17%)	33 (37%)	
Mild paravalvular leak	25 (14%)	5 (5%)	20 (22%)	
Moderate paravalvular leak	2 (1%)	0 (0%)	2 (2%)	
Severe paravalvular leak	1 (1%)	0 (0%)	1 (1%)	

Data presented as mean ± SD or N (%)

*Chi2 value, otherwise it’s a Fischer test or a T-Test value

As per the onset of new conduction abnormalities leading to pacemaker implantation, five devices were implanted in the TF-group (2 in Sapien™ valves and 3 in CoreValves™) and two in the TA-group (*p* = 0.444).

Echocardiographic controls showed similar peak (TA:17 ± 9.4 mmHg, TF:18 ± 9.5, mmHg; *p* = 0.219) and mean transvalvular gradients (TA:9.2 ± 5.1 mmHg, TF:9.7 ± 5.5 mmHg; *p* = 0.563) in both groups. Mild to severe paravalvular leaks were detected more in TF cases (TA:5%, TF:25%, *p* < 0.001) (Table 4). Nevertheless, moderate and severe paravalvular leaks were 0% in TA and 3% in TF patients.

Selected variables were analysed as risk factors for hospital mortality (Table 5), major vascular complications (Table 6) and disabling neurological complications according to the VARC-2 definitions (Table 7).

**Table 5:** Univariate logistical regression analysis for hospital mortality (N:180)

Total	n.0	n.1							
	163	17							
Binary variables	n.0	p.0	n.1	p.1	or	or.sd	or.ci95		p
Transfemoral procedure	81	49.7%	9	52.9%	1.14	1.67	0.42	3.10	0.799
Gender (M)	76	46.6%	6	35.3%	0.62	1.70	0.22	1.77	0.375
COPD	34	20.9%	4	23.5%	1.17	1.83	0.36	3.81	0.798
Vascular disease	81	49.7%	10	58.8%	1.45	1.68	0.52	3.98	0.476
Previous vascular surgery	16	9.8%	1	5.9%	0.57	2.90	0.07	4.62	0.602
Coronary disease	82	50.3%	8	47.1%	0.88	1.67	0.32	2.39	0.799
Previous CABG	25	15.3%	2	11.8%	0.74	2.19	0.16	3.42	0.696
Previous cardiac surgery	35	21.5%	5	29.4%	1.52	1.76	0.50	4.62	0.456
Previous STENT	23	14.1%	5	29.4%	2.54	1.78	0.82	7.87	0.107
Hypertension	108	66.3%	9	52.9%	0.57	1.67	0.21	1.57	0.278
Renal failure	66	40.5%	9	52.9%	1.65	1.67	0.61	4.51	0.326
Previous stroke	17	10.4%	3	17.6%	1.84	1.99	0.48	7.06	0.374
Diabetes	27	16.6%	3	17.6%	1.08	1.95	0.29	4.01	0.909
Pacemaker	19	11.7%	2	11.8%	1.01	2.21	0.21	4.77	0.989
Thorax radiotherapy	14	8.6%	2	11.8%	1.42	2.23	0.29	6.85	0.663
Critical state	11	6.7%	4	23.5%	4.25	1.92	1.19	15.24	0.026
Logistic EuroScore >20%	117	71.8%	12	70.6%	0.94	1.75	0.31	2.83	0.917
LVEF > 50%	91	55.8%	10	62.5%	1.32	1.72	0.46	3.80	0.608
Pulmonary hypertension	95	58.3%	6	35.3%	0.39	1.70	0.14	1.11	0.077
Valve-in-valve	11	6.7%	1	5.9%	0.86	2.94	0.10	7.13	0.892
Vascular complication	8	4.9%	5	29.4%	8.07	1.90	2.28	28.53	0.001
Valve migration	1	0.6%	2	11.8%	21.60	3.50	1.85	252	0.014
Life-threatening bleeding	3	1.8%	4	23.5%	16.41	2.26	3.31	81.29	0.001
Postoperative stroke	2	1.2%	3	17.6%	17.25	2.60	2.66	112	0.003
Pneumonia	4	2.5%	2	11.8%	5.30	2.48	0.90	31.37	0.066
Postoperative acute renal failure	2	1.2%	2	11.8%	10.73	2.82	1.41	81.73	0.022
Postoperative dialysis	1	0.6%	2	11.8%	21.60	3.50	1.85	252	0.014
New pacemaker	6	3.7%	1	5.9%	1.64	3.04	0.19	14.45	0.658
Cardiopulmonary bypass use	1	0.6%	3	17.6%	34.71	3.28	3.38	356	0.003
Early extubation (within 4 h)	118	72.4%	5	29.4%	0.16	1.75	0.05	0.48	0.001
Paravalvular leak (mild to severe)	25	15.4%	3	20.0%	1.37	1.98	0.36	5.21	0.644

OR odds ratio, *OR.SD* odds ratio standard deviation, *OR.CI95* odds ratio 95% confidence interval, *COPD* chronic obstructive pulmonary disease, *CABG* coronary artery bypass graft, *LVEF* left ventricular ejection fraction

**Table 6:** Univariate logistical regression analysis for major vascular complications (N:180)

	n.0	n.1							
Total	167	13							
Binary variables	n.0	p.0	n.1	p.1	or	or.sd	or.ci95		p
Transfemoral procedure	80	47.9%	10	76.9%	3.62	1.97	0.96	13.64	0.057
Gender (M)	78	46.7%	4	30.8%	0.51	1.86	0.15	1.71	0.274
COPD	36	21.6%	2	15.4%	0.66	2.21	0.14	3.12	0.602
Vascular disease	84	50.3%	7	53.8%	1.15	1.78	0.37	3.58	0.806
Previous vascular surgery	15	9.0%	2	15.4%	1.84	2.26	0.37	9.10	0.453
Coronary disease	85	50.9%	5	38.5%	0.60	1.81	0.19	1.92	0.392
Previous CABG	26	15.6%	1	7.7%	0.45	2.89	0.06	3.62	0.455
Previous cardiac surgery	38	22.8%	2	15.4%	0.62	2.20	0.13	2.91	0.542
Hypertension	110	65.9%	7	53.8%	0.60	1.79	0.19	1.88	0.385
Renal failure	72	43.1%	3	23.1%	0.40	1.97	0.11	1.49	0.171
Diabetes	29	17.4%	1	7.7%	0.40	2.89	0.05	3.17	0.383
Pacemaker implantation	20	12.0%	1	7.7%	0.61	2.91	0.08	4.97	0.646
Thorax radiotherapy	14	8.4%	2	15.4%	1.99	2.27	0.40	9.87	0.401
Critical state	14	8.4%	1	7.7%	0.91	2.94	0.11	7.53	0.931
Logistic EuroSCORE >20%	121	72.5%	8	61.5%	0.61	1.81	0.19	1.96	0.404
LVEF > 50%	95	57.2%	6	46.2%	0.64	1.78	0.21	1.99	0.441
Pulmonary hypertension	96	57.5%	5	38.5%	0.46	1.81	0.15	1.47	0.192
Valve-in-valve	11	6.6%	1	7.7%	1.18	2.96	0.14	9.94	0.878
Hospital mortality	12	7.2%	5	38.5%	8.07	1.90	2.28	28.53	0.001
Disabling Stroke	4	2.4%	1	7.7%	3.40	3.18	0.35	32.82	0.291
Pericardial drainage/rethoracotomy for bleeding	4	2.4%	1	7.7%	3.40	3.18	0.35	32.82	0.291
Postoperative acute renal failure	3	1.8%	1	7.7%	4.56	3.30	0.44	47.19	0.204
Postoperative dialysis	2	1.2%	1	7.7%	6.87	3.53	0.58	81.36	0.126
Conversion to sternotomy	1	0.6%	1	7.7%	13.83	4.24	0.81	235	0.069
Cardiopulmonary bypass use	2	1.2%	2	15.4%	15.00	2.85	1.93	116	0.010
Early extubation (within 4 h)	117	70.1%	6	46.2%	0.37	1.79	0.12	1.14	0.084
Paravalvular leak (mild to severe)	26	15.8%	2	16.7%	1.07	2.23	0.22	5.16	0.934

*OR* odds ratio, *OR.SD* odds ratio standard deviation, *OR.CI95* odds ratio 95% confidence interval, *COPD* chronic obstructive pulmonary disease, *CABG* coronary artery bypass graft, *LVEF* left ventricular ejection fraction

**Table 7:** Univariate logistical regression analysis for neurological complications (N:180)

	n.0	n.1							
Total	175	5							
Binary variables	n.0	p.0	n.1	p.1	or	or.sd	or.ci95		p
Transfemoral procedure	87	49.7%	3	60.0%	1.52	2.52	0.25	9.30	0.652
Gender (M)	81	46.3%	1	20.0%	0.29	3.09	0.03	2.65	0.273
COPD	37	21.1%	1	20.0%	0.93	3.11	0.10	8.59	0.951
Vascular disease	88	50.3%	3	60.0%	1.48	2.52	0.24	9.09	0.670
Coronary disease	88	50.3%	2	40.0%	0.66	2.52	0.11	4.04	0.652
Previous CABG	26	14.9%	1	20.0%	1.43	3.12	0.15	13.33	0.752
Previous cardiac surgery	38	21.7%	2	40.0%	2.40	2.54	0.39	14.91	0.346
Previous coronary stenting	27	15.4%	1	20.0%	1.37	3.12	0.15	12.74	0.782
Hypertension	114	65.1%	3	60.0%	0.80	2.53	0.13	4.93	0.812
Previous stroke	19	10.9%	1	20.0%	2.05	3.14	0.22	19.33	0.530
Diabetes	29	16.6%	1	20.0%	1.26	3.12	0.14	11.67	0.840
LVEF > 50%	97	55.7%	4	80.0%	3.18	3.09	0.35	28.99	0.306
Pulmonary hypertension	98	56.0%	3	60.0%	1.18	2.52	0.19	7.23	0.859
Hospital mortality	14	8.0%	3	60.0%	17.25	2.60	2.66	112	0.003
Vascular complication	12	6.9%	1	20.0%	3.40	3.18	0.35	32.82	0.291
Postoperative acute renal failure	3	1.7%	1	20.0%	14.33	3.53	1.21	169	0.035
Postoperative dialysis	2	1.1%	1	20.0%	21.62	3.76	1.61	290	0.020
Early extubation (within 4 h)	121	69.1%	2	40.0%	0.30	2.53	0.05	1.83	0.191

*OR* odds ratio, *OR.SD* odds ratio standard deviation, *OR.CI95* odds ratio 95% confidence interval, *COPD* chronic obstructive pulmonary disease, *CABG* coronary artery bypass grafting, *LVEF* left ventricular ejection fraction

Concerning the hospital mortality, some variables were statistically related to a higher risk of death: critical preoperative state (*p* = 0.026; OR, 4.25; 95% CI, 1.19–15.24), major vascular complications (*p* = 0.001; OR, 8.07; 95% CI, 2.28–28.53), stent-valve migration (*p* = 0.014; OR, 21.6; 95% CI, 1.85–252), life-threatening bleeding (*p* = 0.001; OR, 16.41; 95% CI, 3.31–81.29), disabling stroke (*p* = 0.003; OR, 17.25; 95% CI, 2.66–112), renal failure (*p* = 0.022; OR, 10.73; 95% CI, 1.41–81.73), postoperative dialysis (*p* = 0.014; OR, 21.6; 95% CI, 1.85–252) and need for emergency cardiopulmonary bypass (*p* = 0.003; OR, 34.71; 95% CI, 3.38–356) (Table 5). Early extubation (defined as extubation within 4 h since the end of the index procedure) represents a protective factor against hospital mortality (*p* = 0.001; OR, 0.16; 95% CI, 0.05–0.48).

About risk factors for major vascular complications, use of emergency cardiopulmonary bypass was associated with higher rate of vascular injury (*p* = 0.010; OR, 15; 95% CI, 1.93–116) (Table 6). Concerning risk factors for disabling stroke, postoperative acute renal failure and dialysis were associated with poor neurological outcome (*p* = 0.035; OR, 14.33; 95% CI, 1.21–169; and *p* = 0.020; OR, 21.62; 95% CI, 1.61–290, respectively) (Table 7).

## Discussion

Since the beginning of transcatheter aortic valve therapies, the two main access routes are the transfemoral and the transapical ones, allowing for placement of balloon-expandable and self-expanding valves of different size. However, the allocation to one of these two access routes is still questionable and based, at the time being, on the presence of a severe vascular disease of the aorta or ilio-femoral vessels, or on the possibility of performing the TF case under sedation [18–22].

In our experience, patients allocated to the two groups (all performed under general anaesthesia) showed different comorbidity patterns: patients in the TA-group presented higher prevalence of comorbidities, higher logistic Euro-SCORE and were more often in a preoperative critical state. Thus, according to the fact that all patients with severe vascular disease were automatically included in the TA-group, we faced a TA population carrying a higher risk profile compared to the TF population. However, the hospital mortality rate and neurological outcome were similar.

Hospital mortality of our first TAVI series is in-line with published preliminary results of TA vs TF showing rates ranges between 5% and 15%, without statistical differences between the groups [18–22]. In a retrospective study, Van der Boon reported hospital mortality of 6.4% for TF and 15.7% for TA whereas in a prospective study including thousand TAVI from Schymik and al. the mortality was 6.5% for TF and 6.1% for TA [19, 21]. Murarka and al. also reported promising mortality rates of 4.5% for TF and 5.3% for TA in a retrospective single-centre experience [22]. However, none of these three aforementioned studies was able to demonstrate a statistically significant difference between the two popular approaches.

In our report, neurological outcome showed similar results for TA and TF (stroke = TA:2%, TF:3%, *p* = 1) and we didn’t observe a significant difference. Our results are similar to published data. Schymik described 2.3% of stroke in the TF-group and 1.7% in the TA-group with a non-significant *p*-value between the groups. Other published series show similar conclusions [18–22].

If we consider paravalvular leaks and access-related vascular injuries, we identified a discrepancy in favour for the TA approach. In particular, the mild to severe paravalvular leak incidence (TA:6%, TF:26%, *p* < 0.001) of our patients was similar to the one published in a retrospective study from Greason and co-workers who reported 12% of mild to severe paravalvular leak in TF versus 8.4% in the TA group [18]. Nevertheless, the incidence of moderate-severe PVL only is low in our groups and almost similar between the two population. Murarka and colleagues as well, have observed similar results between TA and TF with 7.6% of moderate-severe paravalvular leaks in TF-group and 7% in TA-group (*p* = 0.999) [22]. The reason for some discrepancies in paravalvular leak rate in the 2 techniques can be due to the more direct and more precise valve positioning during TA cases but this is not proved yet. Moreover, as far as paravalvular leaks are concerned, we have to admit that, in our experience, we have seen a great improvement after the launch of last-generation Sapien™ 3 valve featuring an innovative outer skirt preventing leaks. Therefore, we can speculate that, in the future, the use of this valve (or other stent-valves addressing the issue of paravalvular leaks with improved technologies) will reduce the incidence of clinically-relevant leaks.

Regarding the major access-related vascular complication rate in our group, this was higher in TF patients but the result is not statistically significant (TA:3%, TF:11%, *p* = 0.081). In comparison, if we look at recent publications we can see that the incidence of this complication is more prevalent in TF as well: Schymik reported 17.5% of major vascular complications in TF versus 2.5% in TA (p = <0.0001) [19]. Murarka and colleagues observed similar trend, with 12.1% of major vascular complications in the TF-group versus 0% in the TA-group [22]. New generation devices with low-profile introducer sheaths and small delivery systems will help decreasing the incidence of access site vascular complications in future reports.

As long as the intensive care unit length of stay and extubation time are concerned, in our study these numbers were slightly longer for TA-TAVI patients and this finding is justified by the fact that at the beginning TA patients were all transferred, intubated, to the intensive care unit and then transferred to the intermediate care unit on postoperative day one. On the other hand, since the beginning, complication-free transfemoral TAVI cases were rapidly extubated in the cath-lab and transferred to the intermediate care unit. However, these results are not comparable with other centres as local hospital policies and daily practice can be very different and, sometimes, they can change with acquired experience.

An important point of discussion is the development of new TAVI devices: new generations of stent-valve equipment have more performant designs and low-profile delivery systems assuring lower incidence of paravalvular leak and vascular injury. This development can have a great impact in future reports on TAVI mortality and morbidity allowing for the use of these new devices in mid-risk and younger patients.

Our study presents some limitations. It is a retrospective study describing the hospital outcome of first cases of TA and TF TAVI performed at our Institution with a relatively small number of patients. The population included the preliminary TAVI experience of our hospital and, therefore, a physiologic learning curve can have negatively affected the first-period clinical outcome. The patient-selection process has also changed during the study period as well as the use of new-generation devices.

## Conclusion

In conclusion, this study shows our first TAVI experience and results are similar to published data. Based on our findings, we can confirm that transcatheter techniques are promising and provide good hospital outcomes. Improved surgeons’ and cardiologists’ skills and the advent of last-generation devices will facilitate the stent-valve delivery and will help improving the risk of vascular complication, neurological complication and paravalvular leak, also for use in intermediate-risk patients.

## Abbreviations

3D–CT scan: Three-dimensional Computed Tomography scan
COPD: Chronic obstructive pulmonary disease
LVEF: Left ventricular ejection fraction
SAVR: Standard aortic valve replacement
TA: Transapical
TAVI: Transcatheter aortic valve implantation
TF: Transfemoral
VARC: Valve Academic Research Consortium

## References

[1] Carabello BA (2002). Aortic Stenosis. N Engl J Med.

[2] Nishimura RA, Otto CM, Bonow RO, Carabello BA, Erwin JP, Guyton RA (2014). 2014 AHA/ACC guideline for the management of patients with valvular heart disease: a report of the American College of Cardiology/American Heart Association task force on practice guidelines. J Thorac Cardiovasc Surg.

[3] Czarny MJ, Resar JR (2014). Diagnosis and management of valvular aortic stenosis. Clin Med Insights Cardiol.

[4] Iung B, Baron G, Butchart EG, Delahaye F, Gohlke-Bärwolf C, Levang OW (2003). A prospective survey of patients with valvular heart disease in Europe: the euro heart survey on Valvular heart disease. Eur Heart J.

[5] Ferrari E, von Segesser LK (2010). Transcatheter aortic valve implantation (TAVI): state of the art techniques and future perspectives. Swiss Med Wkly.

[6] Ferrari E, Namasivayam J, Marcucci C, Gronchi F, Berdajs D, Niclauss L, von Segesser LK (2013). Transapical aortic valve replacement in extreme-risk patients: outcome, risk factors and mid-term results. Eur J Cardiothorac Surg.

[7] Leon MB, Smith CR, Mack M, Miller DC, Moses JW, Svensson LG (2010). Transcatheter aortic-valve implantation for aortic stenosis in patients who cannot undergo surgery. N Engl J Med.

[8] Smith CR, Leon MB, Mack MJ, Miller DC, Moses JW, Svensson LG (2011). Transcatheter versus surgical aortic-valve replacement in high-risk patients. N Engl J Med.

[9] Codner P, Assali A, Dvir D, Vaknin-Assa H, Porat E, Shapira Y (2013). Two-year outcomes for patients with severe symptomatic aortic stenosis treated with transcatheter aortic valve implantation. Am J Cardiol.

[10] D’Onofrio A, Salizzoni S, Agrifoglio M, Cota L, Luzi G, Tartara PM (2013). Medium term outcomes of transapical aortic valve implantation: results from the Italian registry of trans-apical aortic valve implantation. Ann Thorac Surg.

[11] Schymik G, Schröfel H, Schymik JS, Wondraschek R, Süselbeck T, Kiefer R (2012). Acute and late outcomes of Transcatheter aortic valve implantation (TAVI) for the treatment of severe symptomatic aortic stenosis in patients at high- and low-surgical risk. J Intervent Cardiol.

[12] Gotzmann M, Korten M, Bojara W, Lindstaedt M, Rahlmann P, Mügge A (2012). Long-term outcome of patients with moderate and severe prosthetic aortic valve regurgitation after transcatheter aortic valve implantation. Am J Cardiol.

[13] Ussia GP, Barbanti M, Cammalleri V, Scarabelli M, Mulè M, Aruta P (2011). Quality-of-life in elderly patients one year after transcatheter aortic valve implantation for severe aortic stenosis. EuroIntervention J Eur Collab Work Group Interv Cardiol Eur Soc Cardiol.

[14] Georgiadou P, Kontodima P, Sbarouni E, Karavolias GK, Smirli A, Xanthos T (2011). Long-term quality of life improvement after transcatheter aortic valve implantation. Am Heart J.

[15] Gonçalves A, Marcos-Alberca P, Almeria C, Feltes G, Hernández-Antolín RA, Rodríguez E (2013). Quality of life improvement at midterm follow-up after transcatheter aortic valve implantation. Int J Cardiol.

[16] Kappetein AP, Head SJ, Généreux P, Piazza N, van Mieghem NM, Blackstone EH (2013). Valve academic research consortium-2. Updated standardized endpoint definitions for transcatheter aortic valve implantation: the valve academic research consortium-2 consensus document. J Thorac Cardiovasc Surg.

[17] Ferrari E, Sulzer C, Marcucci C, Rizzo E, Tozzi P, von Segesser LK (2010). Transapical aortic valve implantation without angiography - proof of concept. Ann Thorac Surg.

[18] Greason KL, Suri RM, Nkomo VT, Rihal CS, Holmes DR, Mathew V (2014). Beyond the learning curve: transapical versus transfemoral transcatheter aortic valve replacement in the treatment of severe aortic valve stenosis. J Card Surg.

[19] Van der Boon RMA, Marcheix B, Tchetche D, Chieffo A, Van Mieghem NM, Dumonteil N (2014). Transapical versus transfemoral aortic valve implantation: a multicenter collaborative study. Ann Thorac Surg.

[20] Gaasch WH, D’Agostino RS (2012). Transcatheter aortic valve implantation: the transfemoral versus the transapical approach. Ann Cardiothorac Surg.

[21] Schymik G, Würth A, Bramlage P, Herbinger T, Heimeshoff M, Pilz L (2014). Long-term results of transapical versus transfemoral TAVI in a real world population of 1000 patients with severe symptomatic aortic stenosis. Circ Cardiovasc Interv.

[22] Murarka S, Lazkani M, Neihaus M, Boggess M, Morris M, Gellert G (2015). Comparison of 30-day outcomes of Transfemoral versus Transapical approach for Transcatheter aortic valve replacement: a single-center US experience. Ann Thorac Surg.

